# Mitigation mechanism of zinc oxide nanoparticles on cadmium toxicity in tomato

**DOI:** 10.3389/fpls.2023.1162372

**Published:** 2023-03-27

**Authors:** Liangliang Sun, Ruting Wang, Qiong Ju, Menglu Xing, Ruishan Li, Weimin Li, Wen Li, Wenying Wang, Yanfang Deng, Jin Xu

**Affiliations:** ^1^ College of Horticulture, Shanxi Agricultural University, Taigu, China; ^2^ College of Life Science, Qinghai Normal University, Xining, China; ^3^ Qinghai Service and Guarantee Center of Qilian Mountains National Park, Xining, China

**Keywords:** ZnO nanoparticles, cadmium stress, cadmium accumulation, flavonoids, alkaloids, amino acids

## Abstract

Cadmium (Cd) pollution seriously reduces the yield and quality of vegetables. Reducing Cd accumulation in vegetables is of great significance for improving food safety and sustainable agricultural development. Here, using tomato as the material, we analyzed the effect of foliar spraying with zinc oxide nanoparticles (ZnO NPs) on Cd accumulation and tolerance in tomato seedlings. Foliar spraying with ZnO NPs improved Cd tolerance by increasing photosynthesis efficiency and antioxidative capacity, while it reduced Cd accumulation by 40.2% in roots and 34.5% in leaves but increased Zn content by 33.9% in roots and 78.6% in leaves. Foliar spraying with ZnO NPs also increased the contents of copper (Cu) and manganese (Mn) in the leaves of Cd-treated tomato seedlings. Subsequent metabonomic analysis showed that ZnO NPs exposure alleviated the fluctuation of metabolic profiling in response to Cd toxicity, and it had a more prominent effect in leaves than in roots. Correlation analysis revealed that several differentially accumulated metabolites were positively or negatively correlated with the growth parameters and physiol-biochemical indexes. We also found that flavonoids and alkaloid metabolites may play an important role in ZnO NP-alleviated Cd toxicity in tomato seedlings. Taken together, the results of this study indicated that foliar spraying with ZnO NPs effectively reduced Cd accumulation in tomato seedlings; moreover, it also reduced oxidative damage, improved the absorption of trace elements, and reduced the metabolic fluctuation caused by Cd toxicity, thus alleviating Cd-induced growth inhibition in tomato seedlings. This study will enable us to better understand how ZnO NPs regulate plant growth and development and provide new insights into the use of ZnO NPs for improving growth and reducing Cd accumulation in vegetables.

## 1 Introduction

Heavy metal pollution seriously affects the safety of agricultural production. Cadmium (Cd) pollution is particularly serious among all kinds of heavy metal pollution. According to a recent survey, 7% of soil contains excessive Cd, and 0.5% is severely contaminated in China. (Wu et al., 2013). Different plants have distinct capacities for Cd uptake, transport and accumulation (Greger, 2004; Zhang et al., 2013). Cd accumulation in plant shoots or fruits is largely determined by the efficiency of root mobilization, uptake and transport of Cd from soil (Chen et al., 2003; Farinati et al., 2010; Jiang et al., 2012; Xue et al., 2012). Cd stress reduces the biomass, fresh weight and dry weight of plants, ultimately affecting crop quality and yield (Wagner, 1993; Rizwan et al., 2017; Wang et al., 2022). Cd toxicity threatens human life and health through the food chain (Wang et al., 2016). Therefore, Cd uptake and accumulation in crops must be controlled to reduce the potential health risks of food consumption. There is an urgent need for innovative solutions to control the problem of heavy metal pollution in crops with the increasing demand for food safety (Lowry et al., 2019).

Tomato (*Solanum lycopersicum*) is an annual herb of the genus *Solanaceae* and is one of the most important vegetable plants in the world (Kimura and Sinha, 2008). Due to its short growth cycle, easy-to-observe phenotype, relatively small genome, and conserved genome structure, tomato is considered to be a representative species of *Solanaceae* (Wang et al., 2018). In recent years, great progress has been made in the development and utilization of tomato, including active substance extraction, tissue culture, functional genomics, comparative genomics, and cloning and expression of functional genes (Jeon et al., 2020). The tomato variety Micro-Tom (MT) has been used as a model plant due to its small size and short life cycle (Shikata and Ezura, 2016). Cd toxicity markedly inhibits growth and development in tomato (Hussain et al., 2019). Reducing Cd accumulation in tomato is of great significance for improving yield and quality and food safety.

Nanomaterials have positive and negative effects on plant growth and development (Lee et al., 2013; Wang et al., 2016; Dimkpa and Bindraban, 2017). Nanotechnology has important application value in the sustainable development of agriculture (Milani et al., 2015; Dimkpa and Bindraban, 2017; Ajmal et al., 2022; Mahamood et al., 2023). Among them, ZnO nanoparticles (ZnO NPs) have been used as an efficient nanofertilizer due to the widespread zinc deficiency in agricultural soils worldwide (Milani et al., 2015; Dimkpa and Bindraban, 2017), and ZnO NPs can also be used as an antibacterial agent (Pranjali et al., 2019). Studies have shown that ZnO NPs have a certain toxic effect on plants (Lee et al., 2013; Wang et al., 2016). ZnO NPs can induce the accumulation of reactive oxygen species (ROS) and subsequent oxidative damage, thereby inhibiting plant growth (Lin and Xing, 2008; Wang et al., 2016; Nair and Chung, 2017; Wan et al., 2019). ZnO NPs can inhibit plant primary root (PR) growth; however, a recovery growth experiment showed that ZnO NP-treated plants recovered faster than Zn^2+^-treated plants (Wan et al., 2019). Several studies have demonstrated the role of ZnO NPs in abiotic and biotic stress responses (Sun et al., 2020; Wan et al., 2020; Zou et al., 2022). ZnO NPs can effectively alleviate chlorosis caused by iron (Fe) deficiency and enhance salt tolerance by reprogramming carbon and nitrogen metabolism and secondary metabolism (Sun et al., 2020; Wan et al., 2020). ZnO NPs and copper oxide nanoparticles (CuO NPs) significantly reduce Cd accumulation in rice and soybean seedlings (Rossi et al., 2018; Ma et al., 2020), but the mechanism is still unclear. Based on this, we investigated the physiological and molecular mechanisms underlying ZnO NP-mediated Cd accumulation in tomato.

## 2 Materials and methods

### 2.1 Plant material and growth conditions

Tomato (*Solanum lycopersicum* cv. Micro-Tom) seeds were sterilized and germinated for approximately 7 d and then transferred to 1/2 Hoagland medium for growth for 2 weeks (Xu et al., 2013). Three-week-old Micro-Tom seedlings were exposed to Cd (5 μM) with or without ZnO NPs (50 mg/L) for 12 d. Specifically, ZnO NPs were sprayed on the leaf surface once every 2 days for a total of 6 times (for 12 d). The culture solution was changed every 3 d during the whole culture process. Plant material was cultured in a climate chamber with a photoperiod of 14 h/10 h (light/dark) and 23 ± 1°C. After 12 d of treatment, the morphological index data were measured and recorded, and plant tissue samples were collected. The leaves were collected from the third to fifth pairs of young leaves at the top of the plant. Samples were quick-frozen in liquid nitrogen after sampling and were stored at -80°C for later use.

### 2.2 ZnO NP stock preparation

In this study, ZnO NPs (purity 99.9%, size 30 ± 10 nm) were purchased from McLean Biotechnology Co., LTD. The average hydrodynamic size and zeta potential for ZnO NPs were 278.23 ± 12.72 nm and 3.09 ± 0.71 mV, respectively. The ZnO NPs were suspended in sterile deionized water (ddH_2_O), stirred for 2 h, and homogenized by ultrasonication at 40 kHz for 60 min until the NPs were evenly distributed as described previously (Zou et al., 2022).

### 2.3 Phenotypic parameters

Plant growth parameters, including plant height, leaf fresh weight (FW), dry weight (DW), primary root (PR) length, chlorophyll content and photosynthetic parameters, were measured after 12 d of Cd, ZnO NPs or combined treatment. Roots were placed in a scanning dish, scanned using a scanner (EPSON Perfection V800 Photo) and analyzed by WINRHIZO (Pro2016A). Chlorophyll content was measured using SPAD 502 (Minolta, Japan). The leaf photosynthetic rate (Pn) was measured using a Li-6800 photosynthesizer (LiCOR, USA). At least three independent biological replicates were performed with 25 plants measured in each treatment group.

### 2.4 Mineral element determination

After treatment, the roots and leaves of tomato seedlings in each group were collected. Samples were soaked in 1 mM EDTA solution for 30 min and then rinsed 5 times with ddH_2_O. Subsequently, the samples were fixed for 15 min at 105°C and dried to constant weight at 70°C. The dried sample (0.3 g) was ground and digested with HNO_3_ according to the method of Sun et al. (2020). The contents of Cd, zinc (Zn), iron (Fe), manganese (Mn) and copper (Cu) were determined by inductively coupled plasma−mass spectrometry (ICP−MS). The experiments are performed in triplicate.

### 2.5 Analysis of antioxidant enzyme activity

Total protein was extracted in potassium phosphate buffer (50 mM, pH 7.8) on ice. After centrifugation (15 min, 15000 rpm, 4°C), the supernatant was removed for determination of superoxide dismutase (SOD), catalase (CAT), and peroxidase (POD) activities. The activities of SOD and CAT were measured by the method described by Xu et al. (2010). POD activity was measured by the method described by Teisseire and Guy (2000).

### 2.6 Metabolomics analysis

Eight root and leaf samples were used for broadly targeted metabolomic analysis (Chong et al., 2018; Mamat et al., 2021). The samples were flash-frozen in liquid nitrogen, ground and extracted, and then tested and partially analyzed by Biotree Biomedical Technology Co., Ltd. (Shanghai, China). Sample testing was performed using an ultra-performance liquid chromatography (UPLC) system that coupled Phenomenex Kinetex columns to a Triple TOF 6600 instrument (QTOF, AB Sciex) (Zou et al., 2022). Each experiment was repeated three times. The process of metabolome data preprocessing includes material screening, noise elimination, normalization and standardization as described by Wang et al. (2022). After data pretreatment, univariate statistical analysis (*t* test) and multivariate statistical analysis (PCA, PLC-DA) were performed to identify the differential accumulation metabolites (DAMs). The screening criteria of DAMs were VIP ≥ 1 and *P* value < 0.05. Kyoto Encyclopedia of Genes and Genomes (KEGG) enrichment analysis and correlation analysis were performed using Metaboanalyst 5.0 (https://www.metaboanalyst.ca/).

### 2.7 Statistical analysis

Three independent biological replicates were used for each experiment in our study. Experimental results are shown as the mean ± standard error (SE). The significance of differences was analyzed using Student’s *t* test (IBM SPSS Statistics 20.0). The asterisk indicates *P* < 0.05. One-way ANOVA with Tukey’s test was used to compare multiple groups. Different lowercase letters represent significant differences at *P* < 0.05.

## 3 Results and discussion

### 3.1 Effects of ZnO NPs on the growth of tomato seedlings under Cd stress

**Figure 1:**
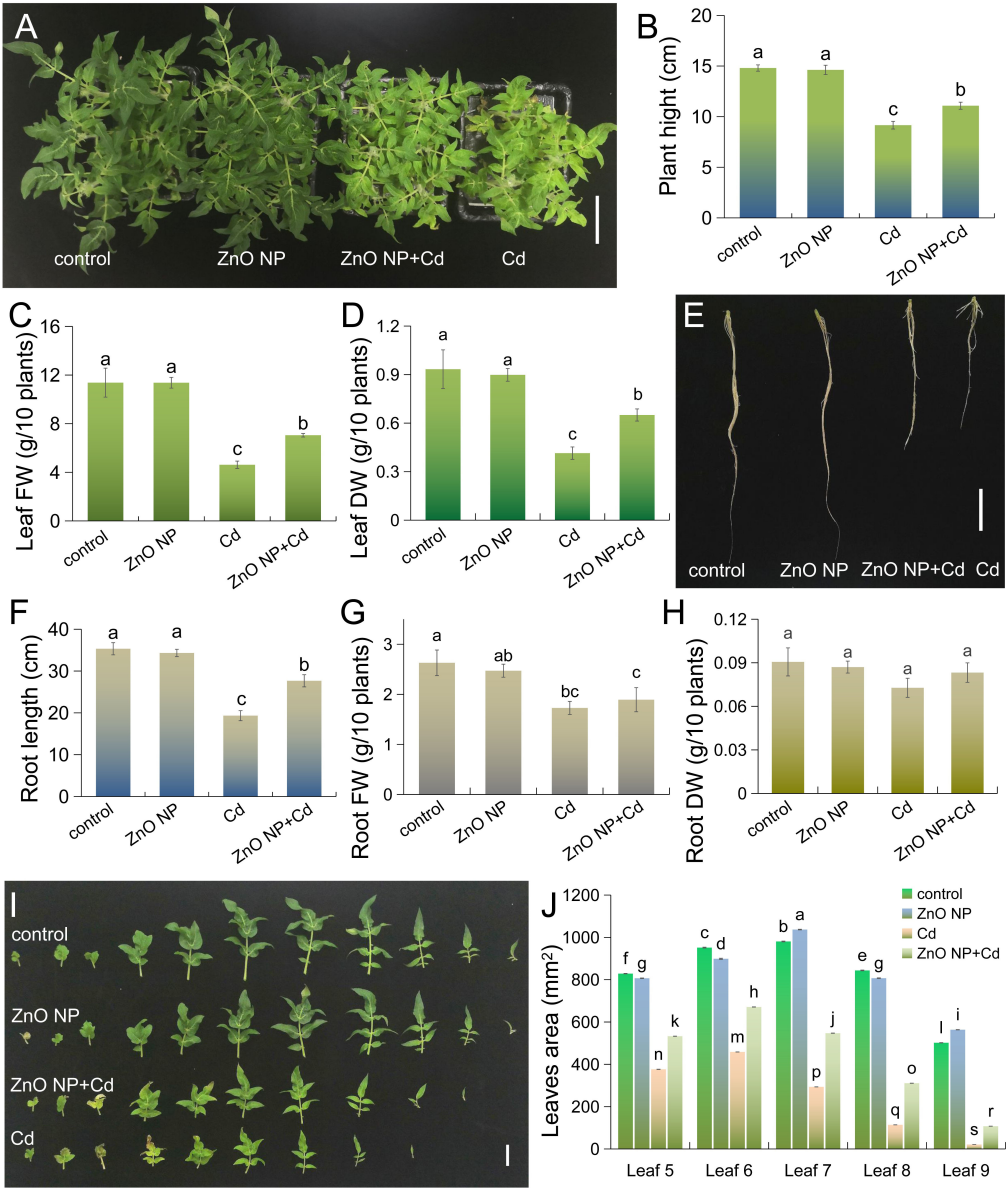
Foliar spraying with 50 mg·L^-1^ ZnO NPs alleviates Cd-mediated growth inhibition in tomato. **(A)** Three-week-old tomato seedlings were transferred to 1/4 strength fresh Hoagland solutions with or without 5 μM CdCl_2_ for 12 days, and the seedlings were foliar sprayed with 50 mg·L^-1^ ZnO NPs (bar = 10 cm); the **(B)** plant height, **(C)** leaf fresh weight (FW), **(D)** leaf dry weight, **(E, F)** root length, **(G)** root FW, **(H)** root DW (bar = 5 cm), and **(I, J)** leaf area of tomato seedlings (bar = 2 cm) were measured. The results shown are the mean ± SE (*n*=3; 15 plants/treatment/replicate), and different letters indicate significant differences (*P* < 0.05 according to Tukey’s test).

We first analyzed the effects of Cd on the growth of tomato seedlings. Cd treatment inhibited plant height, leaf FW and DW, root length, and root FW and DW by 54.7%, 59.4%, 55.6%, 45.3%, 34.3% and 19.8%, respectively (**Figures 1A-H**). This plant growth-inhibiting property of Cd toxicity is also found in plants such as Perilla and mung bean (Aqeel et al., 2021; Wang et al., 2022). This indicates that Cd toxicity has a certain universality in plants. Foliar application of ZnO NPs had no significant effects on tomato plant growth compared to the untreated control (**Figures 1A-H**). Under Cd stress, foliar spraying with ZnO NPs promotes the growth of tomato seedlings. As shown in **Figures 1A-D**, the plant height and leaf FW and DW increased by 20.9%, 52.6% and 56.9%, respectively, in the ‘ZnO NP+Cd’ group compared to Cd treatment alone. This is similar to the inhibitory effect of Cd toxicity on Perilla growth (Wang et al., 2022). Cd stress not only inhibited leaf expansion in tomato seedlings but also reduced leaf number (**Figure 1I**). Foliar application of ZnO NPs had no effect on leaf number and leaf area in tomato compared to the untreated control (**Figure 1I**). Under Cd stress, foliar spraying with ZnO NPs improved leaf growth (**Figures 1I, J**). These results collectively indicated that foliar spraying with ZnO NPs improved Cd tolerance in tomato seedlings. Zou et al. (2022) also obtained similar results in a study of Cd toxicity in tobacco. These results indicate that ZnO NPs have a certain universality in alleviating cadmium toxicity in plants.

### 3.2 Effects of ZnO NPs on photosynthesis in tomato seedlings under Cd stress

**Figure 2:**
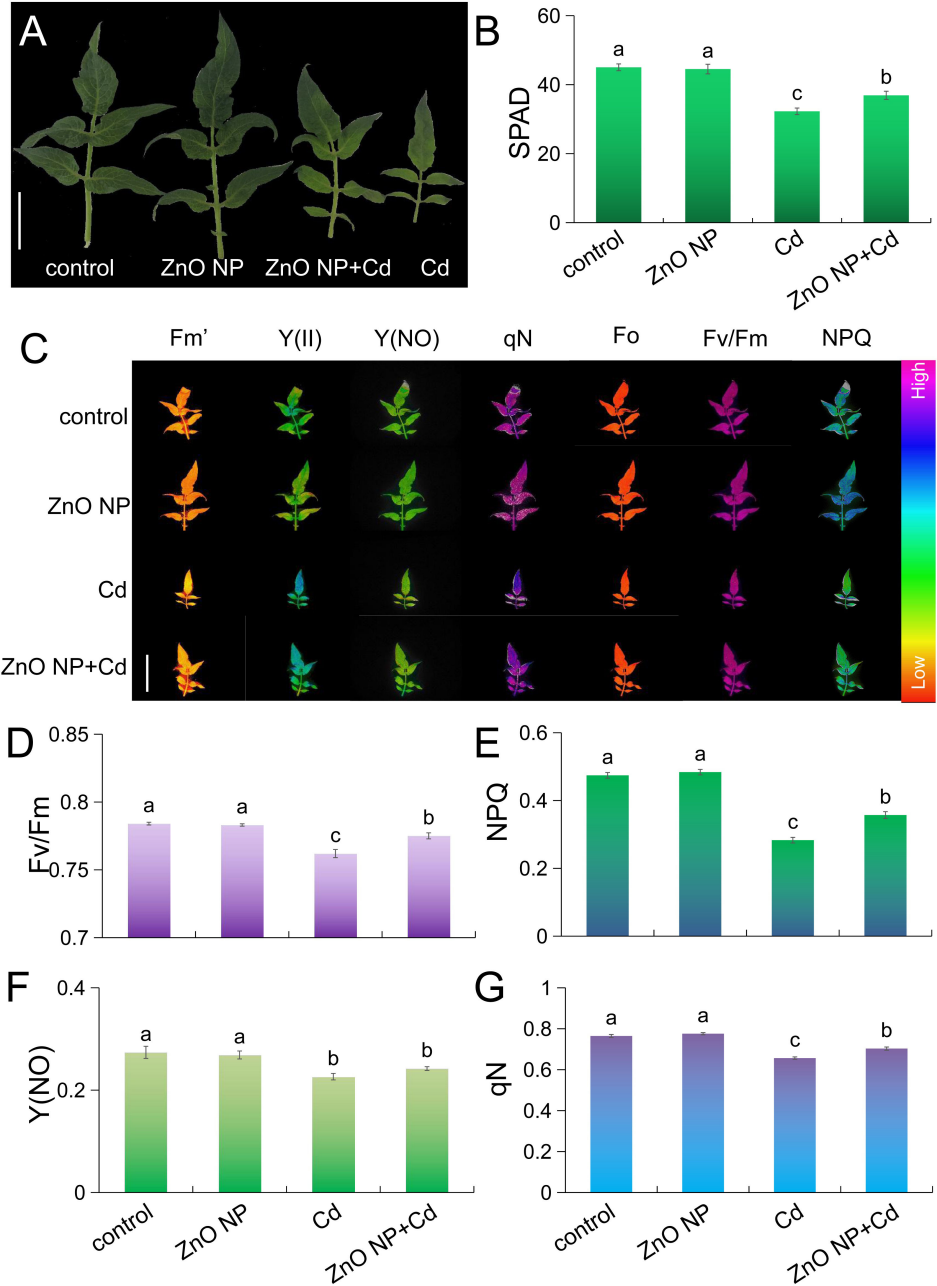
ZnO NPs improve photosynthesis in Cd-treated tomato seedlings. **(A)** Representative leaf images. **(B)** SPAD values (bar = 20 mm). **(C)** Representative chlorophyll fluorescence images and **(D–G)** quantification of Fv/Fm **(D)**, NPQ **(E)**, Y **(F)** and qN **(G)**. Bar = 50 mm. Error bars represent the ± SEs (P < 0.05), and different letters indicate significant differences (*P* < 0.05 according to Tukey’s test).

Cd stress caused etiolation in tomato leaves (**Figure 2A**). Soil and Plant Analyzer Development (SPAD) is a fast and effective method to detect chlorophyll content. We measured chlorophyll contents in tomato leaves using the SPAD method. Cd stress decreased the chlorophyll content by 28.3% in tomato leaves, and this phenomenon is similar to a study on mung bean (Aqeel et al., 2021). ZnO NPs had no adverse effects on chlorophyll content compared to untreated control seedlings (**Figure 2B**). Under Cd stress, foliar spraying with ZnO NPs increased the chlorophyll content by 14.4% in tomato compared with Cd treatment alone (**Figure 2B**). We next determined the photosynthetic parameters in the leaves. Cd stress inhibited chlorophyll fluorescence parameters, such as Fv/Fm (maximum quantum yield), NPQ (nonphotochemical quenching), Y(NO) (lake model quenching parameter) and qN (nonphotochemical fluorescence quenching), in tomato leaves (**Figures 2C**-G). Under Cd stress, foliar spraying with ZnO NPs increased Fv/Fm, NPQ and qN in tomato leaves compared to Cd exposure alone (**Figures 2C-G**). Fv/Fm characterizes the maximum efficiency of “open” photosynthetic system II (PS II) reaction centers capturing excitation energy, and this parameter is used as a sensitive indicator of plant photosynthetic performance (Rod et al., 2012). The decrease in the Fv/Fm value is a typical manifestation of plants under Cd stress (Küpper et al., 2007; Bi et al., 2009; Monteiro et al., 2009; Kummerová et al., 2010). NPQ is a measure of heat dissipation and reflects a mechanism for plant photoprotection, and qN reflects the same photosynthetic state as NPQ (Demmig-Adams et al., 1996; Kato et al., 2003; Adams et al., 2004). Cd stress significantly reduced NPQ, indicating that the photoprotection system was damaged. However, we found that ZnO NPs mitigated this damage caused by Cd stress (**Figures 2C-G**). These results indicated that ZnO NPs improved photosynthesis under Cd stress in tomato seedlings.

### 3.3 Effects of ZnO NPs on antioxidative capacity under Cd stress

**Figure 3:**
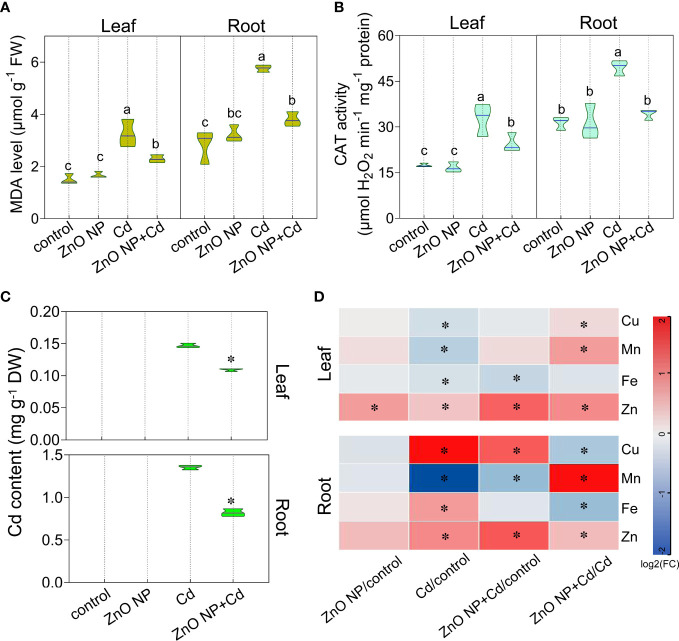
ZnO NPs alleviate Cd-induced oxidative damage and reduce Cd accumulation in tomato seedlings. The **(A)** MDA levels, **(B)** CAT activity, **(C)** Cd contents and **(D)** Cu, Mn, Fe and Zn contents were determined in tomato seedlings. The heatmaps in **(D)** show the log_2_FC values of the different treatments compared with the control **(D)**. The results shown are the mean ± SE (*n*=3; 15 plants/treatment/repeat). Different letters indicate significant differences (*P* < 0.05 according to Tukey’s test). * indicates significantly different values at *P* < 0.05 according to Student’s *t* test.

The degree of oxidative damage and lipid peroxidation level can be evaluated by malondialdehyde (MDA) contents in plants (Barclay and McKersie, 1994). We measured MDA levels in tomato seedlings. Cd stress increased MDA levels by 116.8% and 104.4%, respectively, in the leaves and roots of tomato seedlings (**Figure 3A**). Under Cd stress, foliar spraying with ZnO NPs decreased MDA levels by 29.4% and 34%, respectively, in the leaves and roots compared to the untreated control (**Figure 3A**). We then examined the antioxidative enzyme activities in tomato seedlings and found that foliar spraying with ZnO NPs increased the activity of catalase (CAT), which catalyzes the decomposition of H_2_O_2_ into O_2_ and H_2_O, by 116.6% and 57.8%, respectively, in the leaves and roots of tomato seedlings compared to Cd treatment alone (**Figure 3B**). These results indicated that ZnO NPs alleviated Cd-induced oxidative damage by improving CAT activity in tomato seedlings.

### 3.4 Effects of ZnO NPs on mineral element accumulation in tomato seedlings under Cd stress

Under Cd stress, foliar spraying with ZnO NPs reduced Cd accumulation in tomato seedlings (**Figure 3C**), indicating that ZnO NPs effectively repressed Cd uptake in tomato. Our previous studies have shown that ZnO NP exposure reduces Cd accumulation in tobacco and perilla (Wang et al., 2022; Zou et al., 2022). These results suggest that the effect of ZnO NPs on reducing Cd accumulation is widespread in plants (Hussain et al., 2018; Rizwan et al., 2019a; Rizwan et al., 2019b; Ma et al., 2020; Li et al., 2021; Wang et al., 2022; Zou et al., 2022). In addition, we measured the levels of micronutrients in tomato seedlings. The accumulation of Cu, Mn and Fe in tomato leaves under Cd stress decreased by 16.8%, 30.5% and 14.3%, respectively (**Figure 3D**). Studies have shown that Cd competes with isovalent elements for binding sites or transporters, such as NRAMP (natural resistance-associated macrophage protein), IRT1 (gene encoding a high affinity Fe transporter) and ZIP (zinc-regulated transporter/iron-regulated transporter-related) protein (Guerinot, 2000; Clemens, 2006; Clemens et al., 2013). Therefore, the absorption and transport of mineral elements is disturbed, which affects the accumulation of mineral elements, ultimately inhibiting plant growth (Hédiji et al., 2015). This is consistent with the results of Hédiji et al. (2010). Wu et al. (2004) found that Cd toxicity inhibits the long-distance transport of meal ions from roots to shoots in cotton. Consistent with this result, we found that Cd toxicity increased the accumulation of Cu, Fe and Zn by 270%, 61.5% and 79.4%, respectively, in the roots (**Figure 3D**).

Foliar spraying with ZnO NPs increased the contents of Cu, Mn and Zn by 13%, 60.8% and 78.6%, respectively, in the leaves of tomato under Cd toxicity (**Figure 3D**) and increased the contents of Mn and Zn by 267.8% and 33.9%, respectively, in the roots of tomato under Cd toxicity (**Figure 3D**), suggesting that ZnO NPs improve micronutrient levels under Cd toxicity, thereby improving plant growth. Our previous study revealed that the expression of several metal transporter genes, including *basic HELIX-LOOP-HELIX 38* (*bHLH38*), *bHLH39*, *bHLH100*, *zinc transporter 9* (*ZIP9*), *iron-regulated transporter 1* (*IRT1*), *IRT2*, *niacinamine synthetase 2* (*NAS2*) and *natural resistance associated macrophage protein 4* (*NRAMP4*), was induced, while *NAS3* and *NRAMP1* were repressed by ZnO NPs in *Arabidopsis* (Wan et al., 2019). ZnO NPs increased the contents of Zn and Fe in the leaves and Cu and Mn in the roots of perilla seedlings (Wang et al., 2022), while they also increased Zn contents in the leaves and the contents of Cu, Mg and K in the roots of tobacco (Zou et al., 2022). Altogether, these results indicate that ZnO NPs promote the absorption of mineral elements in plants.

### 3.5 Metabolomics analysis

**Figure 4:**
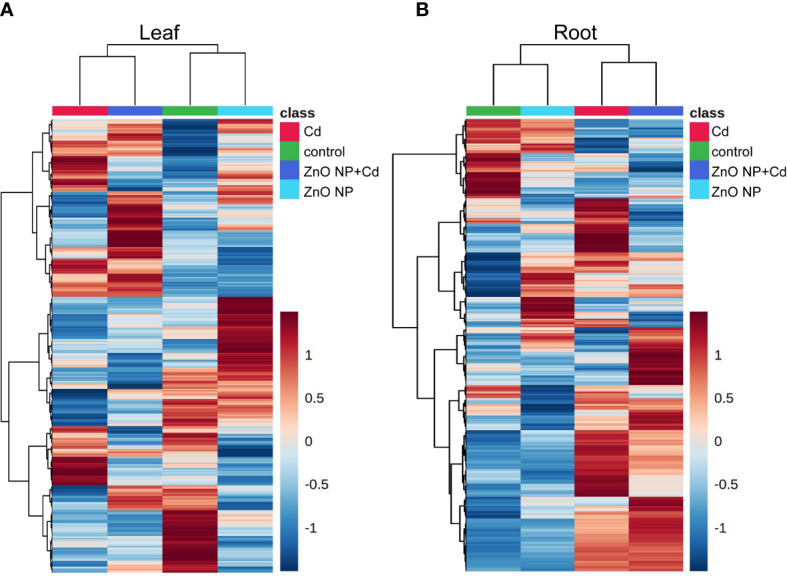
Metabolomic analysis identified the differentially accumulated metabolites in the leaves **(A)** and roots **(B)** of tomato seedlings.

To further elucidate the mechanisms underlying ZnO NP-alleviated Cd toxicity in tomato, a metabonomics analysis was subsequently performed to excavate the differentially accumulated metabolites (DAMs) in the roots and leaves of tomato seedlings. Heatmap analysis showed that the DAMs in the control group were close to those in the ‘ZnO NP’ group, while the DAMs in the ‘Cd’ group were close to those in the ‘ZnO NP+Cd’ group in both the roots and leaves of tomato seedlings (**Figures 4A, B**). PLS-DA showed that compared with the control, the first principal component of the metabolites was significantly separated into the ‘ZnO NP’, ‘Cd’, or ‘ZnO NP+Cd’ group (**Figure S1**), indicating that Cd stress reprogrammed metabolites and that foliar spraying with ZnO NPs changed the levels of metabolites in the roots and leaves of tomato seedlings.

**Figure 5:**
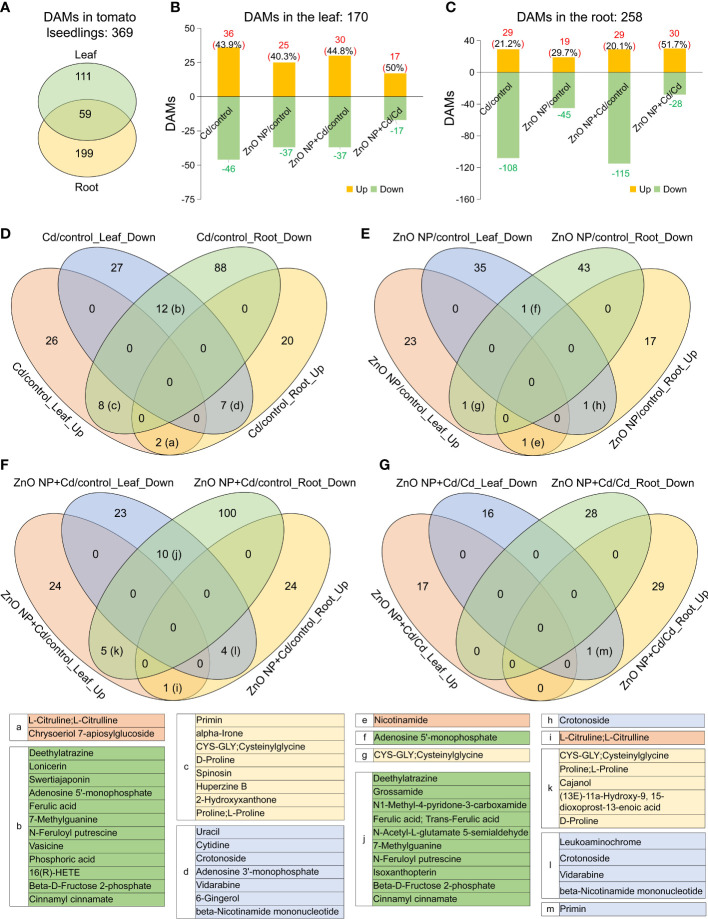
Differentially accumulated metabolites (DAMs) in tomato seedlings. **(A)** Venn diagram analysis showed the overlapping DAMs between leaves and roots. B and C, The number of upregulated and downregulated DAMs in the leaves **(B)** and roots **(C)** of the Cd/control, ZnO NP/control, ZnO NP+Cd/control and ZnO NP+Cd/Cd comparisons. D-G, Venn diagram analysis of DAMs in the leaves and roots of **(D)** Cd/control, **(E)** ZnO NP/control, **(F)** ZnO NP+Cd/control and **(G)** ZnO NP+Cd/Cd comparisons. Up, upregulated metabolites; Down, downregulated metabolites. The tables below list the DAMs in the intersections (a-m) in the Venn diagrams from D to G.

A total of 827 metabolites were identified in tomato seedlings (**Tables S1**). Comparative analysis showed that a total of 170 and 258 DAMs were identified in the leaves and roots of tomato seedlings, respectively (VIP ≧ 1 and *P* value < 0.05) (**Figure 5A**; **Tables S2**). Among them, 59 DAMs were shared between leaves and roots (**Figure 5A**).

A total of 82 DAMs (36 increased and 46 decreased) and 137 DAMs (29 increased and 108 decreased) were identified in the leaves and roots of the Cd/control comparison, respectively (**Figures 5B, C**; **Tables S3**, **S4**); 62 DAMs (25 increased and 37 decreased) and 64 DAMs (19 increased and 45 decreased) were identified in the leaves and roots of the ZnO NP/control comparison, respectively; 67 DAMs (30 increased and 37 decreased) and 144 DAMs (29 increased and 115 decreased) were identified in the leaves and roots of the ZnO NP+Cd/control comparison, respectively; and 37 DAMs (17 increased and 17 decreased) and 58 DAMs (30 increased and 28 decreased) were identified in the leaves and roots of the ZnO NP+Cd/Cd comparison, respectively (**Figures 5B, C**; **Tables S3**, **S4**).

### 3.6 Root and leaf metabolic profiling under cadmium stress or ZnO NP exposure

A total of 36 elevated DAMs (43.9% of the total DAMs) were identified in the leaves, while 29 elevated DAMs (21.2% of the total DAMs) were identified in the roots of the ‘Cd/control’ comparison (**Figures 5B, C**). An additional Venn diagram analysis showed that, in this comparison, two elevated DAMs and 12 reduced DAMs in common were identified in the leaves and roots. In addition, eight metabolites had elevated accumulation in the leaves but reduced accumulation in the roots, and seven DAMs were reduced in the leaves but elevated in the roots (**Figure 5D**). A total of 25 elevated DAMs (40.3% of the total DAMs) in the leaves and 19 elevated DAMs (29.7% of the total DAMs) in the roots were identified in the ZnO NP/control comparison (**Figures 5B, C**). Meanwhile, the Venn diagram analysis showed that, in this comparison, only one common elevated DAM (nicotinamide) and one common reduced DAM (adenosine 5’-monophosphate) were identified in the leaves and roots (**Figure 5E**); one DAM (cysteinylglycine) was elevated in leaves but reduced in roots, whereas one DAM (crotonoside) was reduced in leaves but elevated in roots (**Figure 5E**).

### 3.7 Effects of ZnO NPs on root and leaf metabolomes under Cd stress

In the ZnO NP+Cd/Cd comparison, the accumulation of 30 DAMs was elevated and 37 DAMs were reduced in the leaves, and 29 DAMs were elevated and 115 DAMs were reduced in the roots (**Figures 5B, C**). Venn diagram analysis showed that only one DAM (L-citruline) was elevated and 10 DAMs were reduced in both the leaves and roots (**Figure 5F**). Moreover, the accumulation of 5 DAMs was elevated in the leaves but reduced in the roots, and 4 DAMs were elevated in the roots but reduced in the leaves (**Figure 5F**).

A total of 17 elevated DAMs (50% of the total DAMs) in the leaves and 30 elevated DAMs (51.7% of the total DAMs) in the roots were identified. Venn diagram analysis showed that the accumulation of only one DAM (primin) was reduced in leaves but elevated in roots (**Figure 5G**). Taken together, these results suggest that ZnO NP exposure alleviated the fluctuation of metabolic profiling in response to Cd stress, and it had a more prominent effect in leaves than in roots.

To further elucidate whether ZnO NP exposure can alleviate the metabolic disorder caused by Cd stress, we analyzed the common metabolites in the leaves and roots of tomato seedlings. By comparing the DAMs among the treatment groups, including the Cd/control, ZnO NP/control, ZnO NP+Cd/control comparisons, we found that the levels of flavonoids, phenylpropanoids, alkaloids, amino acid derivatives, nucleotides and their derivatives changed the most in Cd-treated seedlings (**Figure S2**). The accumulation of seven flavonoids, four alkaloids, eight phenylpropanoids, three amino acid derivatives and eight nucleotides and their derivatives increased, while four flavonoids, two alkaloids, five phenylpropanoids and four amino acid derivatives decreased in the leaves of Cd-treated groups (**Figure S2**). Moreover, the accumulation of 12 flavonoids, seven alkaloids, 16 phenylpropanoids, three amino acid derivatives and four nucleotides and their derivatives increased, while one flavonoid, four alkaloids, one amino acid derivative and seven nucleotides and their derivatives decreased in the root (**Figure S2**). Interestingly, we found that ZnO NPs recovered the contents of these metabolites to normal levels in the leaves and roots of Cd-treated seedlings (**Figure S2**). Specifically, the contents of 25 DAMs (including nine flavonoids, five alkaloids, three phenylpropanoids, three amino acid derivatives, five nucleotides and their derivatives) in the leaves and 24 DAMs (including nine flavonoids, five alkaloids, three phenylpropanoids, one amino acid derivative and six nucleotides and their derivatives) in the roots recovered to normal levels after ZnO NP exposure. These results further supported the opinion that ZnO NPs alleviated Cd-induced metabolic disorder in tomato.

### 3.8 Correlation analyses between DAMs and growth parameters

**Figure 6:**
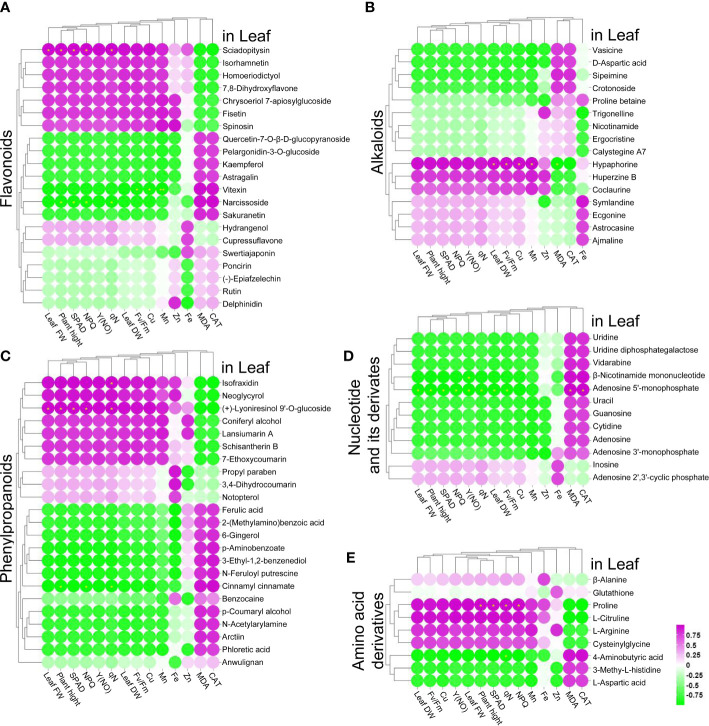
**(A–E)** Correlation analysis of leaf growth parameters with flavonoids **(A)**, phenylpropanoids **(B)**, alkaloids **(C)**, nucleotides and their derivatives **(D)**, and amino acid derivatives **(E)**, * indicates significantly different values at P < 0.05 according to Student’s t test.

The above results indicated that ZnO NPs had a positive effect on alleviating Cd stress. To further explore the relationship between DAMs and the growth and development of tomato seedlings, a correlation analysis was performed between the DAMs and the physiological parameters, including plant height, leaf FW, leaf DW, chlorophyll content, chlorophyll fluorescence, root length, root FW, root DW, antioxidant system, and metal element contents. We found that the accumulation of nine flavonoids, ten phenylpropanoids, seven alkaloids, two nucleotides and their derivatives, and five amino acid derivatives in the leaves was positively correlated with plant height, leaf FW, leaf DW, chlorophyll content, NPQ, Y(NO), qN, Fv/Fm, and the contents of Cu in the leaves, while 12 flavonoids, 13 phenylpropanoids, nine alkaloids, ten nucleotides and their derivatives, and three amino acid derivatives in the leaves exhibited negative correlations with these physiological parameters (**Figures 6A–E**); in contrast, the MDA contents and CAT activity exhibited negative correlations with these DAMs.

In root traits, the accumulation of six flavonoids, six phenylpropanoids, seven alkaloids, seven nucleotides and their derivatives, and three amino acid derivatives in the roots were positively correlated with root length, root FW, root DW and root Mn content, while 20 flavonoids, 22 phenylpropanoids, eight alkaloids, five nucleotides and their derivatives, and seven amino acid derivatives in the roots were negatively correlated with these root physiological parameters (**Figure S3**); the accumulation of 18 flavonoids, 23 phenylpropanoids, eight alkaloids, five nucleotides and their derivatives, and seven amino acid derivatives in the roots were positively correlated with the contents of Cu and Zn, while five flavonoids, six phenylpropanoids, eight alkaloids, seven nucleotides and their derivatives, and two amino acid derivatives in the roots were negatively correlated with the contents of Cu and Zn (**Figure S3**). In addition, the accumulation of 20 flavonoids, 23 phenylpropanoids, ten alkaloids, five nucleotides and their derivatives, and seven amino acid derivatives in the roots were positively correlated, while six flavonoids, seven phenylpropanoids, seven alkaloids, seven nucleotides and their derivatives, and three amino acid derivatives were negatively correlated with root MDA content and CAT activity (**Figure S3**).

Further analysis revealed that several DAMs in the leaves and roots were correlated with plant growth. First, the accumulation of one nucleotide derivative (inosine) and one amino acid derivative (L-citrulline) was positively correlated with the growth parameters (including plant height, leaf FW, leaf DW, root length, root FW and root DW) (**Figures 6**, **S3**). Second, the accumulation of three flavonoids [swertiajaponin, (-)-epiafzelechin and rutin], two phenylpropanoids (ferulic acid and cinnamyl cinnamate), two alkaloids (vasicine and trigonelline) and two nucleotide derivatives (adenosine 5’-monophosphate and guanosine) was negatively correlated with the growth parameters (plant height, leaf FW, leaf DW, root length, root FW and root DW) (**Figures 6**, **S3**). Third, the accumulation of two flavonoids (spinosin and hydrangenol), one phenylpropanoid (coniferyl alcohol), one alkaloid (huperzine B), and two amino acid derivatives (proline and cysteinylglycine) was positively correlated with plant height, leaf FW and leaf DW, while they were negatively correlated with root length, root FW and root DW (**Figures 6**, **S3**). Fourth, the accumulation of one flavonoid (poncirin), two alkaloids (nicotinamide and crotonoside), four nucleotides and their derivatives (uracil, adenosine 3’-monophosphate, β-nicotinamide mononucleotide and cytidine) were negatively correlated with plant height, leaf FW and leaf DW, while they were positively correlated with root length, root FW and root DW (**Figures 6**, **S3**).

**Figure 7:**
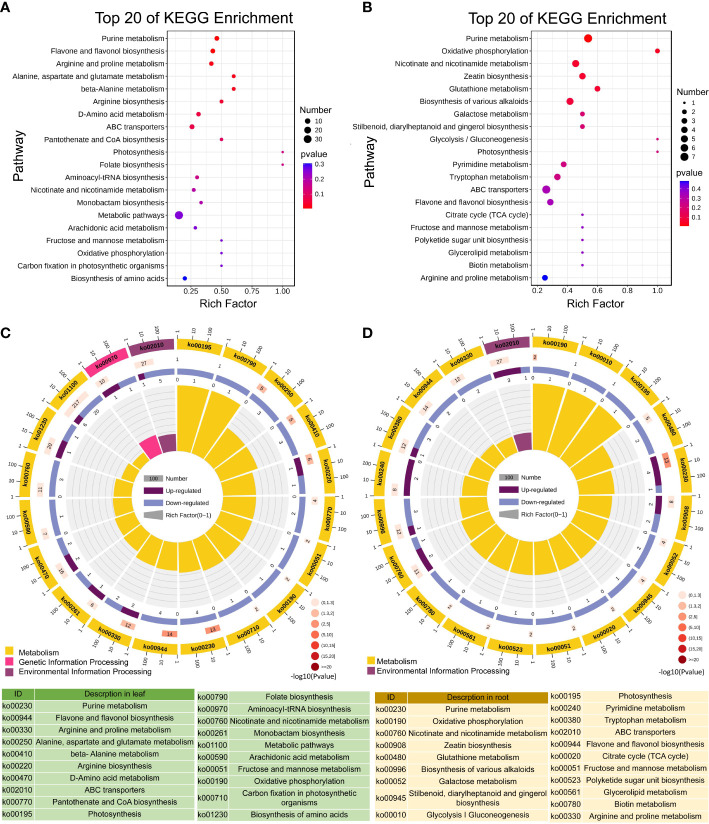
KEGG enrichment analysis of the differentially accumulated metabolites in the leaves and roots of tomato seedlings. Top 20 enriched KEGG pathways of DAMs between the control and Cd-treated leaves **(A, C)**; top 20 enriched KEGG pathways of DAMs between the control and Cd-treated roots **(B, D)**.

Citrulline is ubiquitous in animals, plants, bacteria, and fungi. It is an amino acid that is not involved in protein synthesis but is closely related to arginine metabolism (Moinard and Cynober, 2007; Joshi and Fernie, 2017). In this study, we found that Cd toxicity inhibited L-citrulline accumulation in the leaves and roots of tomato seedlings (**Tables S3**, **S4**). Meanwhile, KEGG metabolic pathway enrichment analysis showed that the arginine and proline metabolism pathways were enriched in the leaves and roots of tomato seedlings under Cd toxicity (**Figures 7A, B**). These results suggest that Cd toxicity inhibits the growth of tomato seedlings by interfering with arginine and proline metabolism. Citrulline is one of the most effective scavengers of hydroxyl free radicals (Yokota et al., 2002). We found that Cd toxicity decreases L-citrulline contents in tomato seedlings, thereby aggravating Cd-induced oxidative damage in plants. Cinnamic acid is a precursor to lignans, polyphenols and substituted derivatives, which are involved in the regulation of various physiological processes in plants (Shuab et al., 2016). Cinnamic acid and its hydroxyl derivatives are synthesized from the aromatic amino acids phenylalanine and tyrosine (Jitareanu et al., 2011). In this study, we found that Cd toxicity induces cinnamate accumulation in leaves and roots (**Tables S3**, **S4**). KEGG analysis showed that the alanine, aspartate and glutamate pathways were enriched in the leaves and that the biosynthesis of the alkaloid pathway was enriched in the roots of tomato seedlings under Cd toxicity (**Figures 7A, B**). These results suggest that Cd stress inhibits the growth of tomato seedlings by interfering with alanine, aspartate and glutamate metabolism and the biosynthesis of alkaloids.

### 3.9 Enrichment analysis of the main metabolite pathways

KEGG enrichment analysis indicated that Cd toxicity regulates metabolic processes, including purine metabolism, flavonol and flavonol biosynthesis, arginine and proline metabolism, amino acid biosynthesis, niacin and niacinamide metabolism, ABC transporters, fructose and mannose metabolism, photosynthesis and oxidative phosphorylation in tomato seedlings (**Figures 7A, B**). The DAMs were mainly enriched in three pathways in the leaves of Cd-treated seedlings, including metabolism, genetic information processing and environmental information processing (**Figure 7C**). In the roots, DAMs were mainly enriched in two pathways: metabolism and environmental information processing (**Figure 7D**).

**Figure 8:**
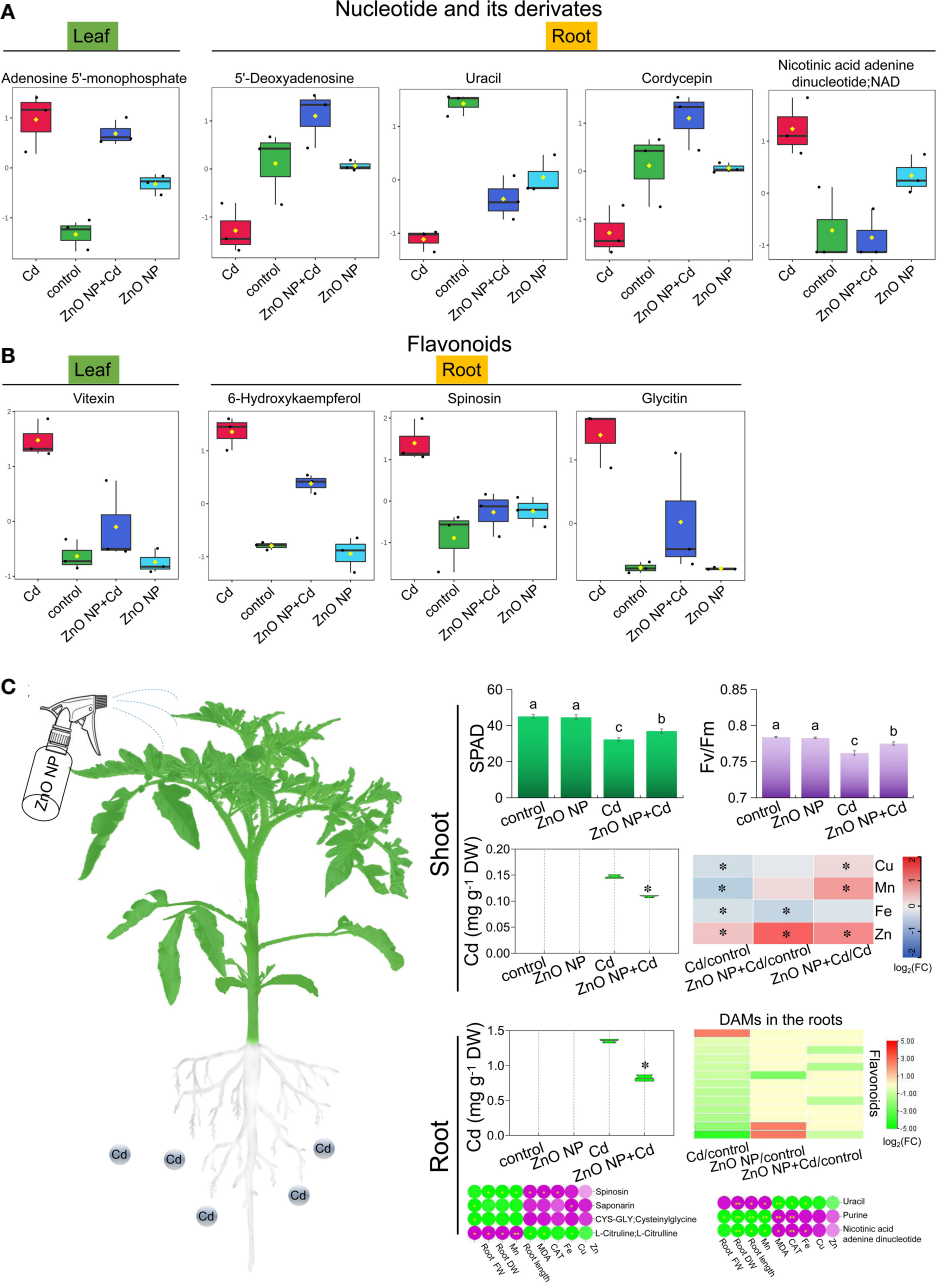
The differentially accumulated **(A)** nucleotide and its derivatives and **(B)** flavonoids in the leaves and roots of tomato seedlings. **(C)** Mechanistic model of ZnO NPs alleviation of Cd stress in tomato seedlings. * indicates significantly different values at P < 0.05, ** indicates significantly different values at P < 0.01 according to Student’s t test.

KEGG analysis revealed that secondary metabolism pathways, including purine metabolism and biosynthesis of flavonoids and flavonols, play important roles in tomato seedlings in response to Cd toxicity (**Figure 7**). Next, we focused on the accumulation of metabolites in purine metabolism and biosynthesis of flavonoids and flavonol pathways and found that Cd stress resulted in a higher accumulation of the nucleotide derivative adenosine 5’-monophosphate in the leaves compared to the control. Compared with Cd stress alone, ZnO NP exposure reduced adenosine 5’-monophosphate levels in the leaves of Cd-treated seedlings (**Figure 8A**). Cd toxicity also reduced the levels of three nucleotide derivatives (5’-deoxyadenosine, uracil and cordycepin) but elevated the level of the nucleotide derivative nicotinic acid adenine dinucleotide in the roots compared to the control (**Figure 8A**). Foliar spraying with ZnO NPs recovered the levels of nicotinic acid adenine dinucleotide in the roots of Cd-treated seedlings (**Figure 8A**). Meanwhile, we found that Cd toxicity resulted in an elevation in the accumulation of the flavonoid compound vitexin in the leaves compared to the control; however, ZnO NP exposure reduced vitexin levels in the leaves of Cd-treated seedlings (**Figure 8B**). Cd toxicity induced an elevation in the accumulation of three flavonoid compounds (6-hydroxykaempferol, spinosin and glycitin) compared to the control, while ZnO NP exposure recovered the levels of these flavonoid compounds in the roots of Cd-treated seedlings (**Figure 8B**).

Secondary metabolites are significantly induced by a variety of abiotic stresses, including light, temperature, salinity and heavy metal stress (Yang et al., 2018; Yeshi et al., 2022). Heavy metal-inducible accumulation of alkaloids, flavonoids and anthocyanins plays an important role in plant stress tolerance responses (Srivastava and Srivastava, 2010; Tan et al., 2021). In this study, we found that the alkaloid biosynthesis pathway was significantly enriched in the roots of tomato under Cd toxicity (**Figure 7B**). Many studies have demonstrated that high concentrations of alkaloids such as cinnamic acid are autotoxic to plants (Ding et al., 2007; Chen et al., 2011). Therefore, excessive alkaloid accumulation caused by Cd stress inhibits plant growth. We found that ZnO NP exposure reduced alkaloid contents to normal levels in Cd-treated seedlings (**Figure S2**), thereby improving plant growth.

Flavonoids play an important role in mediating abiotic and biological stress responses in plants (Wen et al., 2020). In this study, we found that the accumulation of flavonoids decreased significantly in the roots of tomato under Cd toxicity (**Figure S2**). The flavonoid synthesis pathway is a major branch of the phenylpropanoid pathway, which also produces alkaloid compounds, such as lignin and hydroxycinnamic acid, in plants (Vogt, 2010). Therefore, the excessive accumulation of alkaloids consumes upstream substrates, leading to the inhibition of flavonoid biosynthesis, which in turn leads to the reduction of flavonoid accumulation in Cd-treated seedlings (Ahmad and Prasad, 2012). ZnO NP exposure increased the accumulation of most flavonoids to normal levels (**Figure S2**). As an important antioxidant in plants (Ahmad and Prasad, 2012), increased flavonoids improve Cd toxicity tolerance in tomato.

## 4 Conclusion

Cd is one of the most harmful elements with high toxicity to organisms, and Cd pollution threatens sustainable agricultural development and food safety (Godt et al., 2006; Kaur et al., 2017). In this study, we investigated the alleviating effects of ZnO NPs on tomato seedling growth under Cd stress. Our results indicated that foliar spraying with ZnO NPs promoted the growth of tomato seedlings under Cd stress by improving photosynthetic performance and reducing Cd accumulation in tomato seedlings (**Figure 8C**). ZnO NPs effectively inhibit Cd accumulation in plants, thus alleviating the adverse effect of Cd toxicity on plant growth. We found that ZnO NPs significantly induced the uptake and accumulation of Mn and Zn in tomato seedlings (**Figure 3D**). As homovalent elements, Mn and Zn may compete with Cd for metal transporters, resulting in reduced Cd accumulation in plants. This may be an important reason for ZnO NPs to alleviate Cd toxicity in tomato. Furthermore, metabolomic analysis revealed that ZnO NPs reprogrammed plant metabolic pathways, especially alkaloid, amino acid and flavonoid metabolism, in tomato seedlings (**Figure 8C**). Compared with root application (Ma et al., 2020), foliar spraying with NPs can reduce the dosage and cost. This study provides a theoretical basis for using NPs in agricultural production to improve crop yield and quality and provides a new strategy for field management and sustainable agriculture.

## Data availability statement

The original contributions presented in the study are included in the article/**Supplementary Material**. Further inquiries can be directed to the corresponding authors.

## Author contributions

LS, RW and JX designed and supervised the research. LS, RW, MX, and RL performed most experiments. LS, RW, QJ, WML, and MX analyzed and characterized the phenotypes. LS, QJ, and WL analyzed the data, and WW, LS, and JX wrote the manuscript. All authors contributed to the article and approved the submitted version.
