# Supplementary material for: Mitigation mechanism of zinc oxide nanoparticles on cadmium toxicity in tomato
Source: Front Plant Sci. 2023 Mar 27;14:1162372. doi: 10.3389/fpls.2023.1162372 (PMC10083253; doi:10.3389/fpls.2023.1162372)
Supplement: Supplementary file 1 [file DataSheet_1.docx]

Supplementary Material

Mitigation mechanism of ZnO nanoparticles on cadmium toxicity in tomato

**Liangliang Sun**^a^**, Ruting Wang**^a^**, Qiong Ju, Menglu Xing, Ruishan Li, Weimin Li, Wen Li, Wenying Wang*, Yanfang Deng, Jin Xu***

*** Correspondence:** Corresponding Author: xujin@sxau.edu.cn (J. Xu), wangwy0106@163.com (W.Y. Wang).

^a^ These authors contributed equally to this work.

# Supplementary Figures

**
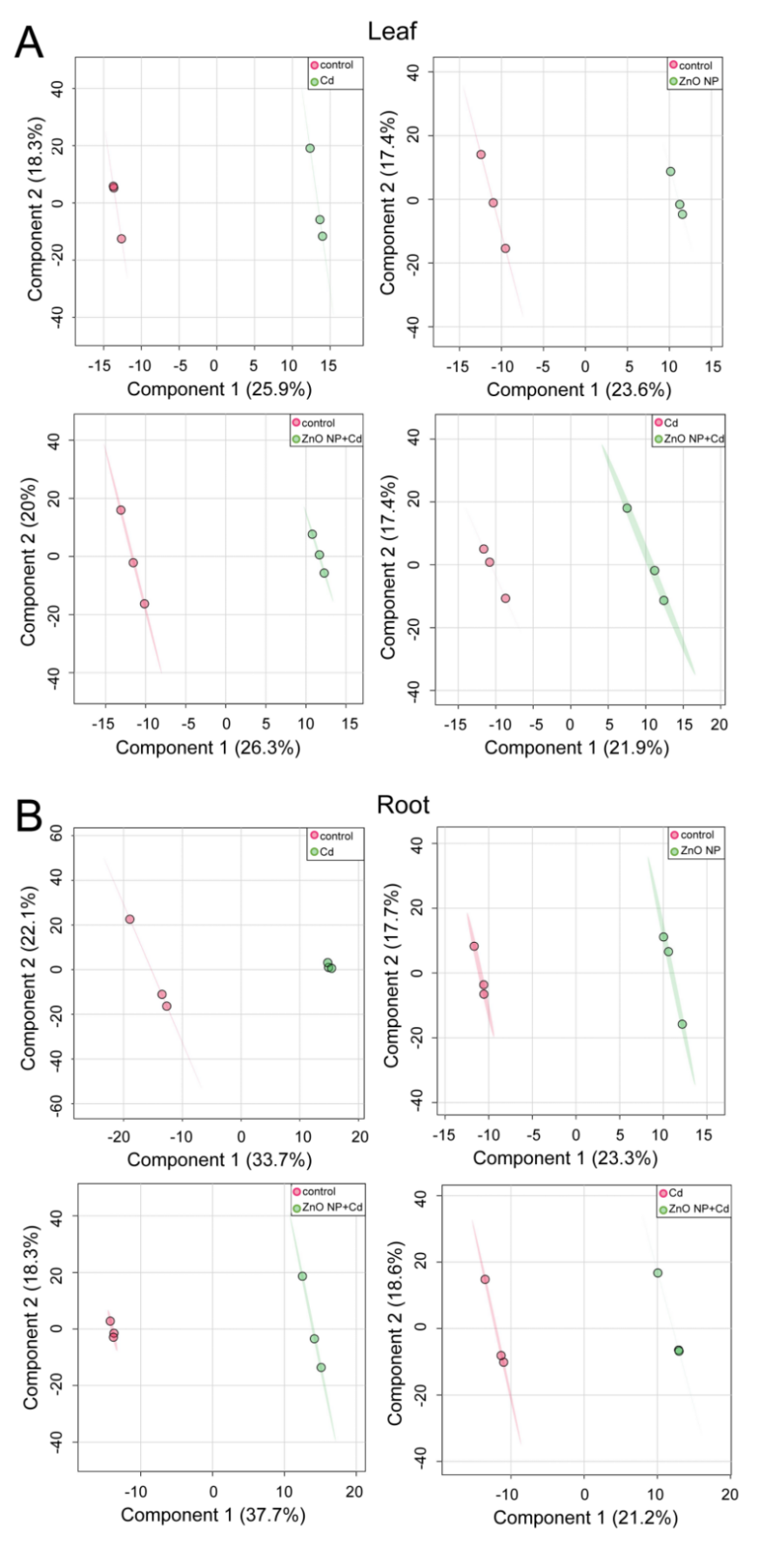
**

**Supplementary Figure 1.** First principal component analysis of metabolites (PLS-DA) in the leaves (A) and roots (B) of tomato seedlings.

**
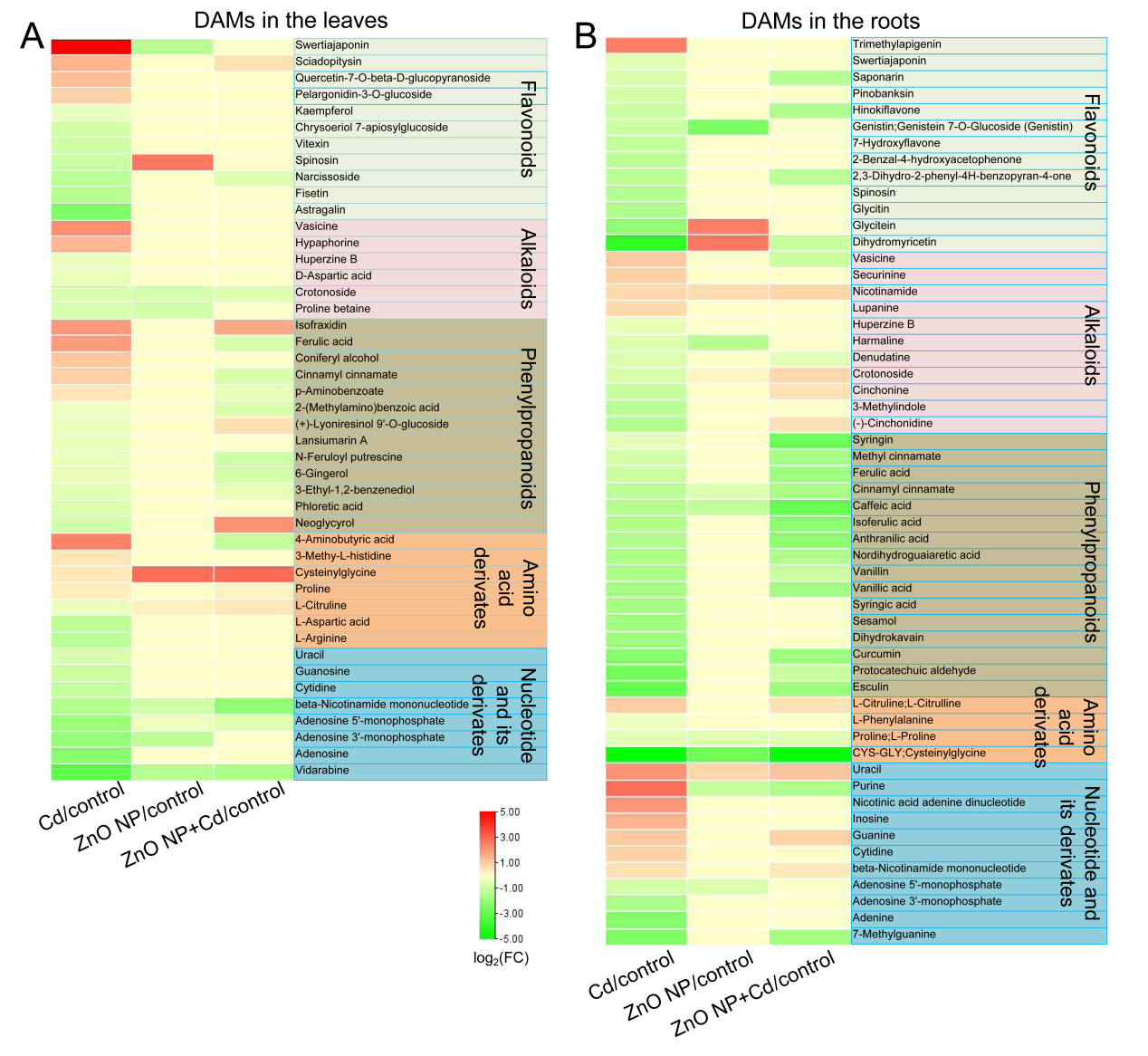
**

**Supplementary Figure 2.** Changes of five species (flavonoids, phenylpropanoids, alkaloids, nucleotide derivatives, and amino acid derivatives) of DAMs in the leaves (A) and roots (B) of tomato seedlings.


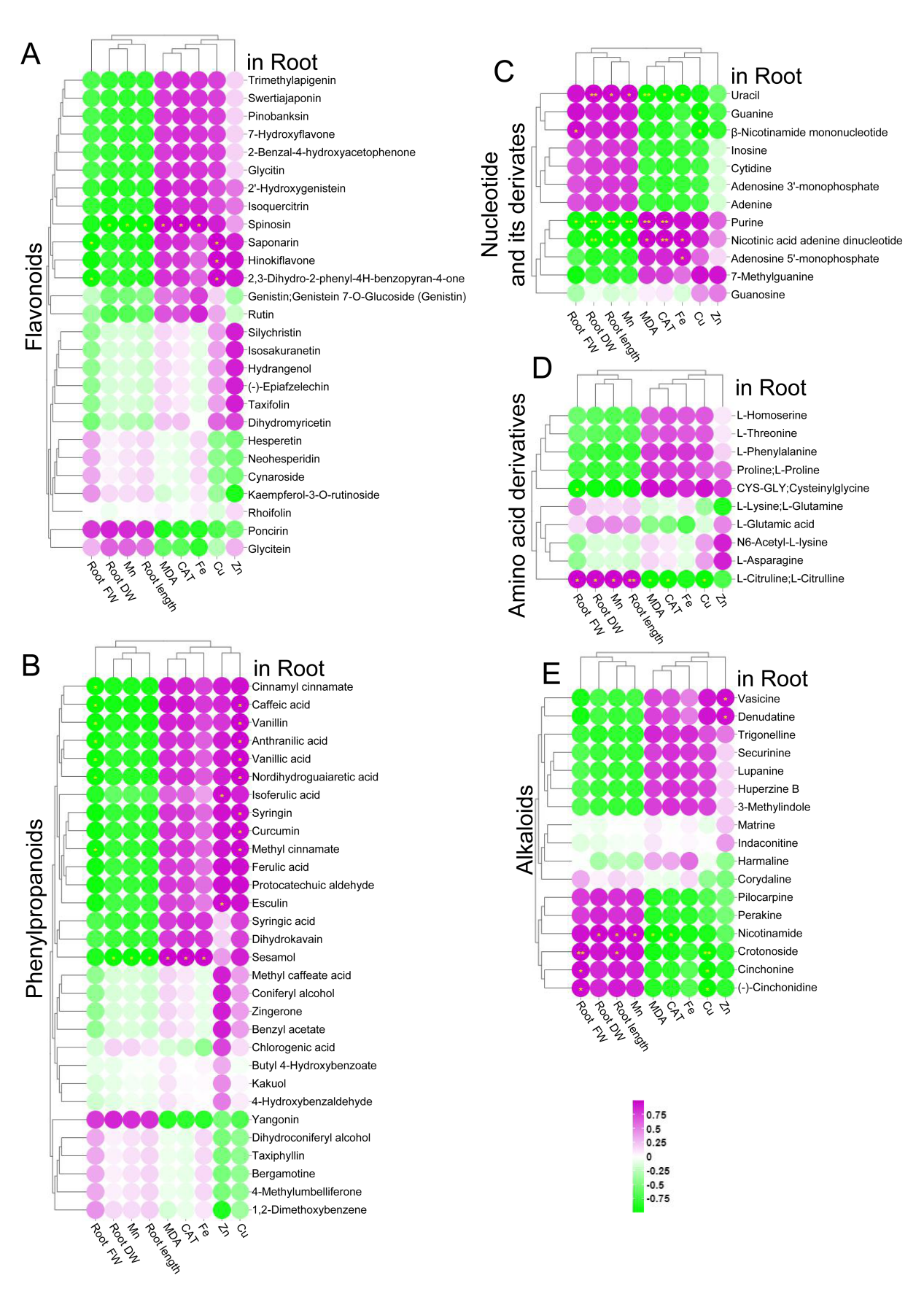


**Supplementary Figure 3.** (A-E) Correlation analysis of root growth parameters with flavonoids (A), phenylpropanoids (B), alkaloids (C), nucleotides and their derivatives (D), and amino acid derivatives (E).

# Supplementary Tables

**Table S1.** The main accumulated metabolites in Tomato (*Solanum lycopersicum cv*. Micro-Tom) seedlings.

**Table S2.** The differentially accumulated metabolites in Tomato (*Solanum lycopersicum cv*. Micro-Tom) seedlings.

**Table S3.** The differentially accumulated metabolites in the leaves of tomato (*Solanum lycopersicum cv*. Micro-Tom) seedlings.

**Table S4.** The differentially accumulated metabolites in the roots of tomato (*Solanum lycopersicum cv*. Micro-Tom) seedlings.
